# Artificial Intelligence: Promise or Pitfalls? A Clinical Vignette of Real-Life ChatGPT Implementation in Perioperative Medicine

**DOI:** 10.1007/s11606-024-08611-2

**Published:** 2024-01-22

**Authors:** Leslie Thienly Ha, Kristen D. Kelley

**Affiliations:** 1 Department of Internal Medicine, University of California, Davis, Davis USA; 2 Sacramento, USA

## INTRODUCTION

Since the release of ChatGPT, a popular new large language model (LLM) developed by OpenAI, there has been speculation about the role of artificial intelligence (AI) in a variety of professions, including many medical fields. A limited number of studies evaluating ChatGPT’s utility for clinical decision support in multiple medical settings have found impressive accuracy, especially as an adjunctive diagnostic tool.^1,2^

While AI has demonstrated high performance in simulated scenarios, there are not many studies investigating its utility in real-world medical decision-making, likely stemming from widespread concerns surrounding potential pitfalls of this technology. One concern is the documented phenomenon of “hallucinations,” in which AI bots provide a confident response that is incorrect or fabricated.^3^ An additional risk posed by medical utilization of this technology is rooted in machine learning’s “garbage in, garbage out” principle: current AI systems are not integrated into the electronic medical record (EMR), thus limiting them to information that the user provides them with. Key elements of clinical information can easily be omitted, leading the AI system to provide recommendations without all necessary information.

We report a case of a 69-year-old man with metastatic prostate cancer who sustained a periprosthetic hip fracture and was additionally found to have bilateral deep venous thromboses (DVT) requiring anticoagulation. He subsequently developed a large hematoma associated with the fracture, positing a complex challenge for the management of perioperative anticoagulation for which the primary orthopedic surgery team utilized ChatGPT to aid in decision-making on timing and type of anticoagulation. This case illustrates many of the concerns around the implementation of ChatGPT as an adjunctive clinical decision support tool, including quality of data input, hallucination, patient autonomy, and privacy.

## CASE

A 69-year-old man with metastatic prostate cancer and bilateral total hip arthroplasties presented after a ground level fall with a right hip periprosthetic fracture. The patient was found to have acute bilateral lower extremity deep venous thromboses (DVT) and started on a therapeutic heparin drip. Given the complex nature of his fracture, surgical intervention was planned for 1 week after admission. However, several days into his hospitalization, the patient developed worsening right thigh pain along with a decrease in hemoglobin from 9.5 to 6.5 g/dL. A computed tomography angiogram demonstrated a large 17.2 cm × 13.9 cm × 45 cm right adductor magnus hematoma.

Given concerns for active bleeding, anticoagulation was held—though not reversed—and the patient was taken for urgent surgical management. The source of bleeding was found to be a torn anterior rectus muscle with difficulty achieving hemostasis intra-operatively, likely related to anticoagulation. He was intra-operatively transfused five units of red blood cells (RBCs), four units of fresh frozen plasma, and two units of platelets. Following the surgical procedure, he was monitored in the surgical intensive care unit, where he required additional RBC and platelet transfusions.

On post-operative day 2, prophylactic enoxaparin was started. The patient continued to experience a slow decline in hemoglobin, requiring additional transfusions though he remained hemodynamically stable. Throughout the hospitalization, the internal medicine (IM) consult service had been following to manage anticoagulation in the setting of acute DVTs balanced against the risk of continued blood loss. Given the post-surgical complications, the IM team recommended holding therapeutic anticoagulation pending stabilization of transfusion requirements. They were additionally considering inferior vena cava (IVC) filter placement in case therapeutic anticoagulation was ultimately not tolerated. The timing of anticoagulation resumption was a complicated decision, which required weighing the patient’s high risk for venous thromboembolism (VTE) against the hemodynamically significant bleed necessitating urgent operative management and multiple transfusions.

The orthopedic surgery team elected to query ChatGPT and inputted the following queries: “*What is the evidence for IVC filter in DVT?*”; “*What are the contraindications for IVC filter*?”; “*What is the risk of PE with bilateral DVT?*”; and “*What is the risk of PE in a patient with DVT who is already on anticoagulation?*” No additional information regarding the patient’s clinical course was provided. ChatGPT replied with summaries of non-specifically cited studies regarding the efficacy and complications associated with IVC filters and risk of pulmonary emboli (PE) in acute DVT (Table 1). Based on the provided information, the orthopedic team concluded that IVC filter complication rates outweighed the risk of developing a PE while on anticoagulation and posited that there was no indication for IVC filter placement. However, it is important to note that the queries did not specify that the patient was on prophylactic rather than therapeutic anticoagulation. The direct responses from ChatGPT along with interpretation of the information by the orthopedic team were documented in the EMR with their plan to continue anticoagulation and defer IVC filter placement.

**Table 1:** Transcript Documenting the Inputted Queries from the Orthopedic Surgery Specialist and the Corresponding ChatGPT Responses

**A**
**Clinician:** What is the evidence for IVC filter in DVT? **ChatGPT**: *Inferior vena cava (IVC) filters are small devices that can be implanted in the IVC vein to prevent blood clots from traveling to the lungs in patients who have deep vein thrombosis (DVT). While IVC filters can be effective in preventing pulmonary embolism (PE), the use of these filters is controversial, and there are potential risks and complications associated with their use.* *Several studies have investigated the effectiveness of IVC filters in preventing PE in patients with DVT. A systematic review and meta-analysis published in the Journal of Vascular Surgery in 2020 found that the use of IVC filters was associated with a significant reduction in the incidence of PE in patients with DVT. However, the study also found that the use of IVC filters was associated with an increased risk of DVT recurrence and filter-related complications such as filter migration, fracture, and perforation.* *Another systematic review and meta-analysis published in the Journal of the American Medical Association in 2019 found that the use of IVC filters did not reduce the risk of PE in patients with DVT and was associated with an increased risk of DVT recurrence and filter-related complications.* ***Based on these findings, the use of IVC filters in patients with DVT is generally reserved for patients who cannot tolerate or have contraindications to anticoagulation therapy, which is the standard treatment for DVT.***
**B**
**Clinician:** What are the contraindications for IVC filter? **ChatGPT**: *There are several contraindications for IVC filter placement, and these include:* *- Allergy or intolerance to the materials used in the filter* *- Inability to access the IVC vein due to anatomical abnormalities or previous surgeries* *- Active bleeding or coagulopathy (a condition that affects the body*’*s ability to form blood clots)* *- Hemodynamic instability (unstable blood pressure or heart rate)* *- Severe pulmonary hypertension (high blood pressure in the lungs)* *- Acute or chronic renal failure (kidney failure)* *- Pregnancy* *- Limited life expectancy or poor prognosis* *- Inability to comply with follow-up care and monitoring after filter placement* *It is important to note that the decision to place an IVC filter should be made after careful consideration of the potential benefits and risks, and in consultation with a qualified healthcare provider. The presence of a contraindication may not always preclude the use of an IVC filter, as each case must be evaluated individually.*
**C**
**Clinician:** What is the risk of PE with bilateral DVT? **ChatGPT:** *The risk of pulmonary embolism (PE) is increased in patients who have bilateral deep vein thrombosis (DVT) compared to those who have DVT in only one leg. This is because blood clots can break off from both legs and travel to the lungs, which can be life-threatening.* *Several studies have investigated the risk of PE in patients with bilateral DVT. One study published in the Journal of Thrombosis and Haemostasis in 2010 found that the incidence of PE was 16.7% in patients with bilateral DVT compared to 6.1% in patients with unilateral DVT. Another study published in the Journal of Vascular Surgery in 2012 found that the incidence of PE was 10.6% in patients with bilateral DVT compared to 3.3% in patients with unilateral DVT.* *These studies highlight the importance of early diagnosis and treatment of DVT, especially in patients who have bilateral DVT. The standard treatment for DVT is anticoagulation therapy, which can help to prevent the formation of new blood clots and the growth of existing ones. In some cases, additional interventions such as the placement of an inferior vena cava (IVC) filter may be considered to prevent blood clots from traveling to the lungs. The decision to use an IVC filter should be made on a case-by-case basis, and the potential benefits and risks should be carefully weighed.*
**D**
**Clinician:** What is the risk of PE in a patient with DVT who is already on anticoagulation? **ChatGPT:** *Anticoagulation therapy is the standard treatment for deep vein thrombosis (DVT), as it can help to prevent the formation of new blood clots and the growth of existing ones. However, even with anticoagulation therapy, there is still a risk of pulmonary embolism (PE) in patients with DVT.* *The risk of PE in a patient with DVT who is already on anticoagulation therapy depends on several factors, including the location and extent of the blood clot, the severity of the underlying medical condition, and the effectiveness of the anticoagulation therapy.* *Several studies have investigated the risk of PE in patients with DVT who are on anticoagulation therapy. One study published in the Journal of Thrombosis and Haemostasis in 2011 found that the incidence of PE was 2.2% in patients with DVT who were on anticoagulation therapy compared to 6.7% in patients who were not on anticoagulation therapy. Another study published in the Journal of the American Medical Association in 2010 found that the incidence of PE was 1.3% in patients with DVT who were on anticoagulation therapy compared to 7.5% in patients who were not on anticoagulation therapy.* *These studies suggest that the risk of PE is lower in patients with DVT who are on anticoagulation therapy compared to those who are not on anticoagulation therapy. However, the risk is not completely eliminated, and patients should continue to be monitored closely for signs and symptoms of PE.*

The IM consultants communicated with the primary team that the patient had not actually been on therapeutic anticoagulation, discussing the consequently misconstrued recommendations from ChatGPT. Over the next 24 h, his hemoglobin stabilized, and the consultants recommended trial of therapeutic enoxaparin. If the patient were to require further transfusions, the team suggested IVC filter placement at that time. Ultimately, he tolerated therapeutic anticoagulation without complications and was transitioned to a direct oral anticoagulant.

## DISCUSSION

The rapid advances in AI technologies have drastically shifted healthcare paradigms across medical specialties, with AI-based systems demonstrating diagnostic acumen in multiple settings.^4–6^ However, despite clear promise, actual AI implementation in daily clinical practice has been limited by concerns including risks of incomplete data input, inaccurate content with hallucination, medicolegal implications, and respect of patient autonomy and privacy. We presented a complex case highlighting many of the concerns regarding use of AI in real-life medical decision-making.

### Insufficient Data Input

In this case, ChatGPT was not provided with key clinical details, including the severity of the bleed and the patient’s hypercoagulable risk factors. The LLM thus provided a general recommendation against use of IVC filter in acute DVT for patients able to tolerate anticoagulation (Table 1A). However, at the time of ChatGPT consultation, it was not yet clear if the patient could tolerate therapeutic anticoagulation given significant intra-operative bleeding, so interpretation of the LLM response to guide against IVC filter placement was premature. ChatGPT’s recommendation was likely influenced by the sparsity of clinical information it received from the inputting user, who may not have been familiar with which clinical factors were most relevant. This highlights the concern of using AI to answer questions that may be outside our scope of practice. We experimented with modifying the prompts provided to ChatGPT, posing a question similar to that shown in Table 1D but specifying that the patient was on prophylactic anticoagulation (Table 2). With this query, the LLM offered a different response, emphasizing persistent risk of PE and noting that this risk is contingent on various patient-specific factors. The LLM did not provide quantifiable risk approximations as it did originally, but spent more time highlighting factors to be considered when assessing thromboembolism risk, similar to the recommendations made by the consulting team. The subtle distinctions between Table 1D and Table 2 suggest that improving the level of detail provided in the prompt results in more appropriately nuanced output. Ultimately, however, the enhanced version of ChatGPT’s response still necessitates a substantial degree of thoughtful interpretation to reach a final clinical decision.

**Table 2:** Transcript Documenting an Experimental Version of the Prompt in Table 1D, Providing Details on the Patient’s Anticoagulation Dosing

**Clinician:** What is the risk of PE in a patient with acute DVT on prophylactic anticoagulation? **ChatGPT:** *In a patient with acute deep vein thrombosis (DVT) who is already on prophylactic anticoagulation, the risk of developing a pulmonary embolism (PE) Is generally lower compared to if the patient were not on any anticoagulation. Prophylactic anticoagulation is prescribed to prevent the extension of the existing DVT and to reduce the risk of new thrombotic events, including the development of a PE.* *However, it’s important to recognize that prophylactic anticoagulation does not completely eliminate the risk of a PE. The risk depends on various factors, including:* ***1. Effectiveness of anticoagulation:**** The choice of anticoagulant, the dosage, and the patient’s adherence to the prescribed regimen all play a role in the effectiveness of prophylactic anticoagulation.* ***2. Underlying risk factors:**** The patient’s overall risk for thromboembolism, including factors such as recent surgery, immobility, cancer, and other medical conditions, influences the likelihood of both DVT and PE.* ***3. Duration of prophylaxis:**** The duration for which prophylactic anticoagulation is prescribed can vary based on the underlying risk factors and the specific clinical scenario. Some patients may require extended prophylaxis.* ***4. Patient-specific factors:**** Individual patient characteristics, such as age, comorbidities and response to anticoagulation, also contribute to the overall risk profile.* *While prophylactic anticoagulation is generally effective in preventing PE in the context of acute DVT, healthcare providers carefully assess each patient’s risk profile and may adjust the treatment plan accordingly. Additionally, regular monitoring for signs of bleeding or other complications is essential.* *Patients on prophylactic anticoagulation should adhere to the prescribed regimen and promptly report any new symptoms or concerns to their healthcare provider. If there are specific questions or concerns about the risk of PE in a patient with acute DVT on prophylactic anticoagulation, it’s recommended to discuss the individual case with the patient’s healthcare team.*	

### Automation Bias and Medicolegal Implications

The potential for insufficient data input coupled with automation bias that may result from overreliance of clinicians on AI can lead to potentially fatal medical decisions made based on inaccuracies. In this case, the AI system was not provided information that the patient was on prophylactic rather than therapeutic anticoagulation. Consequently, the LLM cited studies related to patients on therapeutic anticoagulation, leading the primary team to conclude that current anticoagulation should be continued with no indication for IVC filter (Table 1D). Had the consultant team not pointed out this dosing discrepancy, it is possible that prophylactic dosing would have been continued, exposing the patient to increased VTE risk. This leads to the question of liability and whom is to be held accountable for such errors. As such, these tools should not be independently used to make decisions and physicians must still be held accountable in the review and oversight of generated scripts.

### Hallucination

Another intrinsic limitation of LLMs is hallucination, which refers to errors in AI-generated text that appear semantically plausible but are factually incorrect.^3^ While instances of this did not occur in our case, ChatGPT referenced non-specifically cited journal publications, including the year of publication without titles available for cross-reference (Table 1). In the absence of clear citations coupled with an inability to circumscribe AI bots to peer-validated sources, clinicians are unable to cross-reference evidence provided to confirm validity and relevance. Furthermore, at the time of this case, ChatGPT was limited to datasets up to September 2021; however, OpenAI has since modified its database to include real-time data. While expanding its knowledge base allows access to emerging, up-to-date evidence, this also opens up potential for inclusion of non-validated information that could misinform clinical decisions.

### Patient Autonomy and Privacy

In our case, the documentation of ChatGPT usage was explicit, with transcriptions of the user queries and corresponding chatbot responses attached to the note alongside an independently drafted discussion of the conclusions made by the clinician. The incorporation of AI chatbots as a third-party component of a supposedly shared medical decision-making process between patient and provider poses the question of how to respect patient autonomy. Maximizing transparency in how systems such as ChatGPT are factored into clinical decision-making through explicit documentation is crucial to clearly delineate what data elements were obtained from the AI versus which were formulated by a clinician. Furthermore, by aiming to depict the most comprehensive clinical picture, clinicians are at risk of disclosing too much and compromising patient privacy. LLMs cannot truly be made HIPAA compliant due to AI’s inherent ability to infer sensitive information even with de-identified data.^7^ Thus, utilization of these chatbots necessitates a careful balance between providing adequate clinical information for safe medical decision-making while maintaining privacy.

## CONCLUSION

Overall, while we agree that utilization of AI and LLM by clinicians as an adjunctive tool to specialty consultation may facilitate thoughtful, multi-directional discussions, we caution against overreliance upon this tool for medical decision-making, especially in its current form. As described, there are many limitations of AI and LLMs, and real-world patients have significantly more intricacies than easily communicated via free text to a chatbot. In this case, while the medical team did provide the same overall recommendation of not placing an IVC filter, these conclusions were reached for different reasons. It is easy to imagine a scenario in which the LLM could lead providers to a dangerous clinical decision in the absence of adequate clinical context. Equally, there is a risk of providing too much data that can compromise patient confidentiality and lead to unauthorized use of patient information by third parties, necessitating novel legislative measures beyond existing health privacy laws, which are insufficient to guard against AI-related privacy issues.

At the time of this case, our health system had not yet issued any formal policy regarding use of AI. However, since then, the institution has released recommendations to refrain from providing sensitive data, including de-identified patient information, to AI systems until there is more information on these systems and their appropriate usage. It is clear that further observational studies involving the direct implementation of AI into real-time clinical decision-making are needed to elucidate additional consequences of using this technology and guide providers in pivoting their utilization accordingly. Achieving a more nuanced and comprehensive understanding of these fallibilities will facilitate physician education and training on the usage of these models as well as empower us to engage in the careful regulation and oversight necessary for the actualization of ChatGPT’s potential in daily clinical practice. The pitfalls outlined in this vignette illustrate that ChatGPT in its current state poses more potential harm than benefit, and will require not only further studies but potentially even a redesign of the system with safeguards to mitigate against healthcare-specific pitfalls that could compromise patient safety and privacy.
